# A unique maternal and placental galectin signature upon SARS-CoV-2 infection suggests galectin-1 as a key alarmin at the maternal–fetal interface

**DOI:** 10.3389/fimmu.2023.1196395

**Published:** 2023-07-05

**Authors:** Fangqi Zhao, Ann-Christin Tallarek, Yiru Wang, Yiran Xie, Anke Diemert, Alice Lu-Culligan, Pavithra Vijayakumar, Enrico Kittmann, Christopher Urbschat, Juan Bayo, Petra C. Arck, Shelli F. Farhadian, Gabriela S. Dveksler, Mariana G. Garcia, Sandra M. Blois

**Affiliations:** ^1^ Department of Obstetrics and Fetal Medicine, University Medical Center Hamburg-Eppendorf, Hamburg, Germany; ^2^ Department of Immunobiology, Yale School of Medicine, New Haven, CT, United States; ^3^ Department of Obstetrics, Gynecology, and Reproductive Sciences, Yale School of Medicine, New Haven, CT, United States; ^4^ Gene Therapy Laboratory, Instituto de Investigaciones en Medicina Traslacional, Consejo Nacional de Investigaciones Científicas y Técnicas (CONICET) - Universidad Austral, Buenos Aires, Argentina; ^5^ Section of Infectious Diseases, Department of Internal Medicine, Yale School of Medicine, Yale University, New Haven, CT, United States; ^6^ Department of Pathology, Uniformed Services University of the Health Sciences, Bethesda, MD, United States

**Keywords:** galectin-1, galectin-3, galectin-9, galectin-7, PSG1, SARS-CoV-2, COVID-19

## Abstract

The severe acute respiratory syndrome coronavirus 2 (SARS-CoV-2) pandemic imposed a risk of infection and disease in pregnant women and neonates. Successful pregnancy requires a fine-tuned regulation of the maternal immune system to accommodate the growing fetus and to protect the mother from infection. Galectins, a family of β-galactoside–binding proteins, modulate immune and inflammatory processes and have been recognized as critical factors in reproductive orchestration, including maternal immune adaptation in pregnancy. Pregnancy-specific glycoprotein 1 (PSG1) is a recently identified gal-1 ligand at the maternal–fetal interface, which may facilitate a successful pregnancy. Several studies suggest that galectins are involved in the immune response in SARS-CoV-2–infected patients. However, the galectins and PSG1 signature upon SARS-CoV-2 infection and vaccination during pregnancy remain unclear. In the present study, we examined the maternal circulating levels of galectins (gal-1, gal-3, gal-7, and gal-9) and PSG1 in pregnant women infected with SARS-CoV-2 before vaccination or uninfected women who were vaccinated against SARS-CoV-2 and correlated their expression with different pregnancy parameters. SARS-CoV-2 infection or vaccination during pregnancy provoked an increase in maternal gal-1 circulating levels. On the other hand, levels of PSG1 were only augmented upon SARS-CoV-2 infection. A healthy pregnancy is associated with a positive correlation between gal-1 concentrations and gal-3 or gal-9; however, no correlation was observed between these lectins during SARS-CoV-2 infection. Transcriptome analysis of the placenta showed that gal-1, gal-3, and several PSG and glycoenzymes responsible for the synthesis of gal-1-binding glycotopes (such as linkage-specific N-acetyl-glucosaminyltransferases (MGATs)) are upregulated in pregnant women infected with SARS-CoV-2. Collectively, our findings identify a dynamically regulated “galectin-specific signature” that accompanies the SARS-CoV-2 infection and vaccination in pregnancy, and they highlight a potentially significant role for gal-1 as a key pregnancy protective alarmin during virus infection.

## 1 Introduction

Severe acute respiratory syndrome coronavirus 2 (SARS-CoV-2) infection poses a particular risk to pregnant women and their neonates, in which pregnant women have higher rates of severe coronavirus disease 2019 (COVID-19) disease than non-pregnant adults (1). Pregnant women experience immunologic and physiologic changes, such as swelling of the respiratory tract and restricted lung expansion, that make them less tolerant to viral respiratory infections, especially in the last trimester (2, 3). Maternal SARS-CoV-2 infection affects pregnancy outcomes, with increased incidence rates of intensive care admission, invasive ventilation, maternal death, iatrogenic preterm birth, and stillbirth (4–12). Furthermore, comorbidities such as obesity, diabetes, heart disease, advanced maternal age, or a lack of vaccination increase the risk of severe COVID-19 symptoms (3, 11). A recent study revealed no association between the gestational age at infection and COVID-19 morbidity and mortality, suggesting that a previously reported increase in morbidity and mortality in the third trimester may be attributable to other gestational age–affected variables for which adjustment was made in this study (4, 13). Therefore, there is a continuing challenge for clinicians to manage the SARS-CoV-2 infection during pregnancy (1, 9). In this regard, SARS-CoV-2 vaccines have shown efficacy in preventing symptomatic maternal illness and are considered safe for both the mother and infant (8, 9, 14–16). SARS-CoV-2 antibodies have been documented in umbilical cord blood and breast milk after maternal vaccination, suggesting protection for the fetus (9, 14, 17–23). On the basis of the growing evidence that supports the safety and efficacy of COVID-19 vaccination in pregnancy (8, 9, 15–25), most countries recommend full COVID-19 vaccination, regardless of pregnancy trimester.

Galectins are a family of endogenous carbohydrate-binding proteins characterized by a unique sequence motif in their carbohydrate recognition domain (CRD), with the ability to bind β-galactosidase residues (6, 26–28). Galectins are classified into three major types: prototype, which contain one CRD and form homodimers (e.g., gal-1 and gal-7); chimera containing a C-terminal CRD and a proline- and glycine-rich N terminal tail that mediate their oligomerization (gal-3); and tandem repeats that have two different CRDs in tandem connected by a linker of up to 70 amino acids (e.g., gal-9). Galectins contribute to healthy gestation by modulating multiple immune and inflammatory processes (6). In normal pregnancy, circulating maternal gal-1 levels increase from the first trimester, peaking during the second trimester, and remain similar until term (28). Being less abundant, upregulation of gal-3, gal-7, and gal-9 levels in maternal circulation occurs mainly in the second trimester (26, 27). In the extracellular compartment, galectins bind to the glycans decorating glycoproteins. One identified gal-1 ligand at the maternal–fetal interface is pregnancy-specific glycoprotein 1 (PSG1); glycans in the N- and A2 domains of PSG1 mediate the interaction between these two molecules (29). PSGs belong to the carcinoembryonic antigen family within the immunoglobulin (Ig) superfamily and are secreted to the maternal circulation by trophoblast cells (30, 31). PSG1 is one of 10 PSGs and is considered one of the most abundant trophoblastic proteins in maternal circulation during the third trimester of pregnancy (31). PSG1 interacts with soluble and membrane-bound ligands and participates in processes required for successful pregnancy (29). Functional studies with recombinant PSG1 showed that this protein has pro-angiogenic activity; inhibits the interaction of fibrinogen with platelets; and regulates extravillous trophoblast adhesion, migration, and invasion (32–34). In addition, PSG1 activates the latent form of the anti-inflammatory cytokines Transforming growth factor β (TGF-β1) and (TGF-β2) by binding to the respective latent associated peptides contributing to the establishment of maternal immune tolerance (30, 35, 36). Dynamic changes in PSG1 maternal circulation levels during virus infection remain unexplored.

A growing body of clinical data suggests that the cytokine release syndrome is one of the main reasons for the high mortality observed in COVID-19 patients (37). Changes in the cytokine profile of pregnant women correlate with the clinical severity of patients with COVID-19 (10, 38). Recent studies revealed that circulating gal-1, gal-3, and gal-9 levels are increased in non-pregnant patients with COVID-19 (39–43). Levels of gal-3 and gal-9 were reported to be upregulated only in patients with severe disease (40, 41), raising the possibility that circulating gal-3 or gal-9 can be valuable biomarkers for severe pneumonia in patients with COVID-19 (44, 45). Interestingly, exogenous gal-9 administration during acute SARS-CoV-2 infection has been reported to increase the survival rate inducing a robust innate and adaptive immune response in mice (46). More recently, an inhaled gal-3 inhibitor has been tested as a potential therapy for COVID-19 pneumonitis (47). However, despite considerable progress in dissecting the functions of individual members of the galectin family, there is no comprehensive study of the galectin signature in the maternal circulation and placenta following SARS-CoV-2 infection and vaccination during pregnancy.

Therefore, we aimed to determine the galectin fingerprint in the maternal and placental compartment during COVID-19 disease and SARS-CoV-2 vaccination in pregnancy. Our findings indicate that gal-1 is uniquely upregulated at the maternal circulation during SARS-CoV-2 infection and following vaccination. These results underscore the importance of gal-1 as a key pregnancy protective alarmin during SARS-CoV-2 infection. In addition, we observed that the concentration of PSG1, a gal-1 ligand, was increased following infection, providing the first indication of potential regulation of this trophoblast-derived protein in response to insults to maternal health.

## 2 Material and methods

### 2.1 Participants and data collection: human subjects for the study of SARS-CoV-2 infection and vaccination during pregnancy

For the current study, we used two pregnancy cohorts as follows.

#### 2.1.1 PRINCE cohort

The PRINCE study is a longitudinal prospective cohort of pregnant women and their children located at the University Medical Center Hamburg-Eppendorf. This cohort aims to identify prenatal factors influencing maternal and children’s future immune development and health. Women were included if they were at least 18 years old, were not expecting twins, and were between 12th and 14th ± 6 weeks of pregnancy with regular checking up by a specialized obstetrician. They were excluded if the pregnancy was conceived medically assisted, an autoimmune disorder was diagnosed, or fetal pathologies were observed. The PRINCE COVID cohort was established in March 2020 and recruited pregnant women infected with SARS-CoV-2 at any point during pregnancy. The third cohort, PRINCE VACCINE, included SARS-CoV-2–negative women vaccinated twice within an interval of 6 weeks with 30 μg of BNT162b2 messenger RNA COVID-19 vaccine during pregnancy. All study subjects signed informed consent forms, and the ethics committee of the Hamburg Chamber of Physicians (Ärztekammer Hamburg) approved the study protocol under the registration numbers PV3694 (PRINCE), PV 7312-4710 (PRINCE COVID), and 2021-10647-BO-ff (PRINCE VACCINE).

#### 2.1.2 Yale IMPACT cohort

In the Yale study, women who were in labor at Yale New Haven Hospital from 27 March 2020 to 1 June 2020 and tested positive for SARS-CoV-2 by nasopharyngeal (NP) swab Quantitative reverse transcription polymerase chain reaction (RT-qPCR) were recruited to the Yale IMPACT study. These participants provided informed consent for research studies of donated placental tissue and blood. The placenta and blood of the control group were selected from SARS-CoV-2–uninfected women (as determined by negative RT-qPCR testing of NP swab) at Yale New Haven Hospital with matching maternal age, gestational age, and maternal comorbidities to the COVID-19 placental cases, recruited during the same months as the SARS-CoV-2–infected participants. They also provided informed consent to serve as uninfected controls for transcriptomic studies. The study was approved by the Yale Institutional Review Board (protocol #2000027690).

### 2.2 Determination of circulating galectins and PSG1 levels

For both PRINCE and Yale IMPACT studies, blood was collected in serum separator tubes, and serum was aliquoted and stored immediately at −80°C for further analysis. Human galectins and PSG1 levels were measured in the serum by enzyme-linked immunosorbent assay (ELISA) as previously described (26, 28). Briefly, Corning® 96-Well High-Binding (Fisher Scientific) was coated overnight with either polyclonal anti-human gal-1, gal-3, gal-7, gal-9, or anti-PSG1 antibodies (500-P210; PeproTech, AF 1154, 842118, AF 2045, and DY6799-05; R&D Systems, USA, respectively) and washed with washing buffer. Plates were blocked with 1%–2% Bovine serum albumin (BSA) in Phosphate-buffered saline (PBS). Individual wells were incubated with serial dilutions of galectins or PSG1 standards or serum samples for 1–2 h at room temperature (RT). Wells were washed and incubated with biotinylated polyclonal anti-human galectins or PSG1 antibodies (500-P210; PeproTech, AF 1154, 842118, AF 2045, and DY6799-05; R&D Systems, USA, respectively). Plates were washed three to six times and incubated with horseradish peroxidase–conjugated streptavidin (189733; Calbiochem, USA). After three to eight additional washes, a colorimetric reaction was developed with the 3,3,5,5′-tetramethyl benzidine. The reaction was stopped by adding one volume of 4 N H_2_SO_4_, and absorbance at 450 nm was recorded.

### 2.3 RNA sequencing data analysis

Bulk RNA sequencing analysis was performed on placental RNA sequencing data previously described (48). For the present study, FASTQ files were normalized with Kallisto v0.46.188 using the “-b 100 and -t 20” options to obtain transcript abundances in transcript per million (TPM). Expression data for our selected genes were downloaded, and differential expression analysis was performed using the Welch’s t-test. Genes were considered differentially expressed if *P*-value < 0.05 (**Supplementary Table 1**). The fold change in gene expression was represented with Z-score after being calculated from TPM values and visualized as a heatmap.

### 2.4 Statistical analyses

GraphPad PRISM version 9 and R Statistical Software were utilized for statistical analysis. Correlations between the serum galectin levels and the clinical parameters were conducted using Spearman’s correlation analysis. Statistical parameters, including sample sizes and dispersion, are reported in the figures and their legends. Statistical difference between two groups was determined by an unpaired, two tailed *t-*test. One-way analysis of variance with Bonferroni or Dunn’s multiple comparisons was used to compare different groups. If data did not meet test prerequisites, equivalent non-parametric tests were utilized. Data were considered to be statistically significant if *P* ≤ 0.05.

## 3 Results

### 3.1 Clinical characteristics of patients in the PRINCE COVID cohort

To explore the impact of SARS-CoV-2 infection or vaccination in the maternal galectin profile, women from the PRINCE COVID cohort were grouped as follows: pregnant women who were tested positive for SARS-CoV-2 by NP swab qRT-PCR any time during pregnancy, matched control pregnant women tested negative for SARS-CoV-2 during the whole gestation, pregnant women who received two doses of vaccination, and matched control vaccinated non-pregnant women. Most of SARS-CoV-2 infections (75%, 15 of 20) were diagnosed during the second trimester of pregnancy, and all these patients had a negative viral test (NP swab qRT-PCR) at the time of delivery. Of the 30 patients, 24 (80%) received their first vaccination dose in the second trimester. Maternal and neonatal characteristics are summarized in **Table 1**. No severe COVID-19 disease (intensive care unit stay or administration of supplemental oxygen required) was observed in the PRINCE COVID cohort. All pregnancies included in the cohort resulted in live births, with no complications related to SARS-CoV-2 infection or vaccination. There were no significant differences among healthy pregnant women, pregnant women with COVID-19, and pregnant women who were vaccinated in terms of maternal age, body mass index (BMI), gestational age, mode of delivery, neonatal outcomes, or comorbidities.

**Table 1:** Clinical characteristics of the PRINCE cohorts.

	Pregnant healthy controls (n=20)	Pregnant COVID- 19 patients (n=20)	Pregnant vaccinated women (n=30)	Non-pregnant vaccinated controls (n=30)
Age: mean (range)	33.6 (27-42)	31.6 (22-40)	34.0 (26-39)	32.3 (22-42)
BMI: mean (SD)	22.1 (1.81)	22.0 (2.36)	26.6 (4.75)	24.1 (4.93)
Gestational week: median (range)	39 + 0 (37-41)	40 + 5 (38-41)	39 + 5 (38-42)	
Mode of delivery (% CS)	10.0%	20.0%	22%	
Sex of infant (% male)	60%	45.5%	50%	
Gravidity: median (range)	1 (1-2)	2 (1-6)	1 (1-3)	
Parity: median (range)	1 (0-2)	1 (0-4)	1 (0-3)	
Neonatal Apgar, 5 min:	10 (7-10)	10 (9-10)	10 (9-10)	
Infant body weight (g): mean (SD)	3513.5 (427.9)	3525.2 (478.2)	3396.5 (287.2)	
Comorbidities				
Hypertension	0	0	0	3.4%
Preeclampsia	0	5%	0	0
Diabetes	0	0	0	0
COVID-19 features				
COVID-19 symptoms at time of delivery (%)		75%		
severe COVID-19		0		

BMI, body mass index previously pregnancy in pregnant healthy controls and pregnant COVID-19 patients and at the time of the sample collection in pregnant vaccinated patients; CS, cesarean section.

### 3.2 Maternal circulating gal-1 levels increase with SARS-CoV-2 infection or vaccination in pregnant women

**Figure 1:**
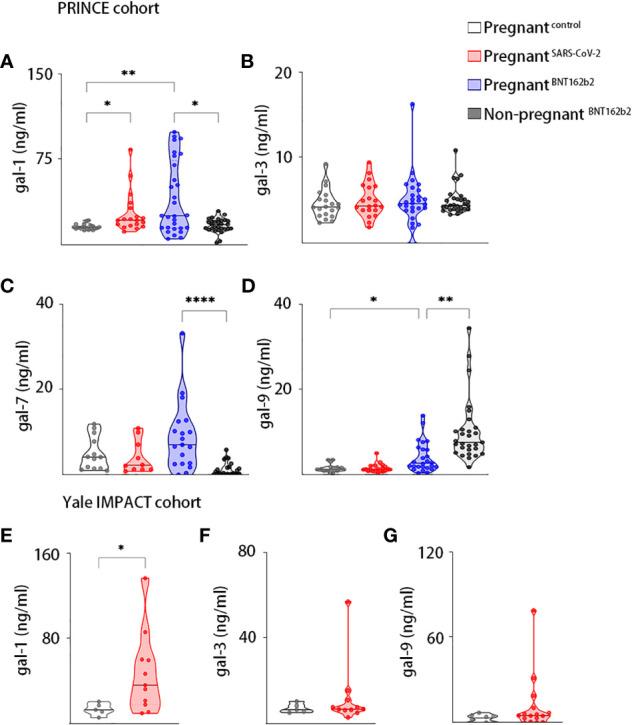
Galectins dynamics upon SARS-Cov-2 infection or vaccination during pregnancy. Maternal circulating levels of gal-1 **(A)**, gal-3 **(B)**, gal-7 **(C)**, and gal-9 **(D)** in the PRINCE cohort analyzed by ELISA in healthy pregnant women (pregnant^control^, n = 20), pregnant women infected by SARS-CoV-2 (pregnant^SARS-CoV-2^, n = 20), pregnant women vaccinated against SARS-CoV-2 (pregnant^BNT162b2^, n = 30), and non-pregnant vaccinated women (non-pregnant^BNT162b2^, n = 30). Circulating levels of gal-1 **(E)**, gal-3 **(F)**, and gal-9 **(G)** in the Yale IMPACT cohort analyzed by ELISA in healthy pregnant women (pregnant^control^, n = 5) or pregnant women infected by SARS-CoV-2 (pregnant^SARS-CoV-2^, n = 11). *P < 0.05, **P < 0.01, and ****P < 0.0001 as analyzed by Kruskal–Wallis test or Welch’s t-test. In all figures, circulating levels of galectins and PSG1 were determined in triplicate for each serum sample.

We first measured the circulating gal-1, gal-3, gal-7, and gal-9 levels of SARS-CoV-2–infected pregnant women, uninfected and unvaccinated healthy pregnant women, pregnant women who were vaccinated against SARS-CoV-2, and healthy vaccinated women who were not pregnant in the PRINCE cohort. Our results showed that circulating gal-1 levels were increased in pregnant women with antecedent SARS-CoV-2 infection (*P*-value = 0.0185) and those who were vaccinated against SARS-CoV-2 (*P*-value = 0.0054) compared with control healthy unvaccinated pregnant patients (**Figure 1A**). We further observed that vaccinated women displayed higher levels of circulating gal-1 (*P*-value = 0.0313) if they were pregnant (**Figure 1A**), whereas they had similar levels of gal-3 in all the compared groups (**Figure 1B**). In addition, no differences were observed in the circulating levels of gal-7 and gal-9 between healthy pregnant women and the SARS-CoV-2 previously infected pregnant women (**Figures 1C, D** **)**. Increased gal-9 levels in circulation were noticed in vaccinated pregnant women compared with the ones who were not vaccinated (*P*-value = 0.0243, **Figure 1D**). Furthermore, pregnant vaccinated women presented higher serum levels of gal-7 (*P*-value < 0.0001) and lower concentrations of gal-9 (*P*-value = 0.0015) compared with non-pregnant vaccinated group (**Figures 1C, D**).

### 3.3 Yale IMPACT cohort studies

We next sought to assess the galectins/PSG1 signature in acute SARS-CoV-2 infection during pregnancy. **Table 2** describes the clinical features of the Yale IMPACT cohort, which were previously described (48). In addition, SARS-CoV-2–uninfected women (as determined by negative RT-qPCR testing of NP swab) were recruited as control. Among SARS-CoV-2–infected women, 45.5% (5 of 11) had symptomatic COVID-19, and two cases of severe COVID-19 disease required supplemental oxygen administration. All pregnancies resulted in live births, with a median Apgar score of 9 (range, 4–9). No significant differences existed between cases and matched controls for maternal age, gestational age, demographics, and neonatal outcomes. However, the groups differed in rates of gestational hypertension (45.5% in infected versus 0% in uninfected), preeclampsia (36.4% versus 0%), and diabetes (18.2% versus 0%).

**Table 2:** Clinical characteristics of the Yale IMPACT cohort.

	Pregnant healthy controls (n=5)	Pregnant COVID-19 patients (n=11)
Age: mean (range)	34.4 (30-42)	30.1 (20-40)
Gestational week: median (range)	39 + 2 (39-40)	39 + 5 (37-41)
Mode of delivery (% CS)	100%	45.5%
Sex of infant (% male)	60%	64%
Gravidity: median (range)	3 (1-4)	2 (1-4)
Parity: median (range)	1 (0-3)	1 (0-2)
Neonatal Apgar, 1 min: median (range)	9 (4-9)	9 (7-9)
Comorbidities		
Hypertension	0	45.5%
Preeclampsia	0	36.4%
Diabetes	0	18.2%
COVID-19 features		
COVID-19 symptoms at time of delivery (%)		45.5%
severe COVID-19		18.2%
SARS-CoV-2 PCR testing of NP swab CT value: median (range)		32.3 (17.1-32.5)

CS, cesarean section; CT, cycle threshold.

In the Yale IMPACT cohort, we analyzed the circulating levels of gal-1, gal-3, and gal-9 in SARS-CoV-2–infected pregnant women and healthy pregnant controls. We found significantly higher levels of gal-1 in the serum of patients with COVID-19 compared with healthy pregnant controls (*P*-value = 0.0156, **Figure 1E**). However, no significant differences were observed in the circulating levels of gal-3 and gal-9 among both groups (**Figures 1F, G**).

### 3.4 Correlation of maternal gal-1, gal-3, and gal-9 levels is altered following SARS-CoV-2 infection

**Figure 2:**
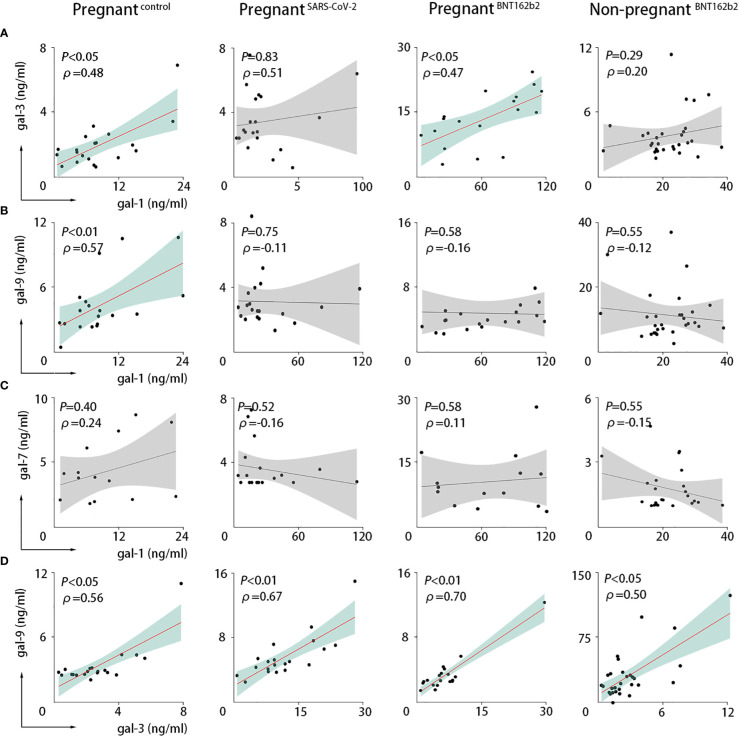
SARS-CoV-2 infection or vaccination altered the maternal circulating levels. Correlations in PRINCE cohort between serum values of gal-1 and gal-3 **(A)**, gal-1 and gal-9 **(B)**, gal-1 and gal-7 **(C)**, or gal-3 and gal-9 **(D)**. Pregnant^control^, healthy pregnant women (n = 20); pregnant^SARS-CoV-2^, pregnant women infected by SARS-CoV-2 (n = 20); pregnant^BNT162b2^, pregnant women vaccinated against SARS-CoV-2 (n = 30); non-pregnant^BNT162b2^, non-pregnant vaccinated women (n = 30). The Spearman correlation coefficient (ρ) is shown. *P <* 0.05 was considered statistically significant (green) and *P >* 0.05 not significant (gray) as analyzed with the Spearman statistical test.

We performed correlation analysis to explore the dynamics of galectin levels at the maternal circulation. During a healthy pregnancy, we found a significant positive correlation between gal-1 and gal-3 [*P*-value = 0.0331, Spearman correlation coefficient (ρ) = 0.478; **Figure 2A**] and between gal-1 and gal-9 (*P*-value = 0.0083, ρ = 0.571; **Figure 2B**). SARS-CoV-2 infection resulted in the loss of the observed correlation of gal-1 with both gal-3 and gal-9 (**Figures 2A, B**). On the other hand, vaccination only compromised the correlation between gal-1 and gal-9 (**Figure 2B**), whereas the correlation between gal-1 and gal-3 was maintained (*P*-value = 0.0433, ρ = 0.468; **Figure 2A**). No significant correlation was found between gal-1 and gal-7 in any of the analyzed groups (**Figure 2C**). Our results also demonstrated that gal-3 was positively associated with the levels of gal-9 in healthy pregnant women (*P*-value = 0.0103, ρ = 0.562; **Figure 2D**) and that this correlation was still present in the SARS-CoV-2–infected (*P*-value = 0.0010, ρ = 0.666; **Figure 2D**) and the SARS-CoV-2 vaccinated individuals (*P*-value = 0.0013, ρ = 0.704; **Figure 2D**).

Further correlation studies between galectins and clinical features in the PRINCE cohort were performed by analyzing general clinical parameters, including BMI, age, parity, gestational week of delivery, and the birth weight and height of the neonate. In addition, for each experimental group, we considered the time of infection (gestational week and trimester) and the gestational week in which women received the first and second doses of vaccination. Our results showed that the BMI of the patients was positively associated with gal-1 levels in maternal circulation (*P* < 0.0001, ρ = 0.439; **Table 3**) and gal-3 levels (*P* = 0.0241, ρ = 0.240; **Table 3**), and the age of the patients inversely correlated with gal-9 levels (*P* = 0.0141, ρ = −0.267; **Table 3**). In addition, when we analyzed the correlation between galectins and clinical parameters in the Yale IMPACT cohort, our results showed that gal-1 was negatively associated with the parity of the pregnant women (*P* = 0.0029, ρ = −0.844; **Table 4**).

**Table 3:** PRINCE cohort correlations between galectins and clinical parameters.

	gal-1 (ng/ml)		gal-3 (ng/ml)		gal-7 (ng/ml)		gal-9 (ng/ml)	
	*P*	*ρ*	*P*	*ρ*	*P*	*ρ*	*P*	*ρ*
BMI	<0.001	0.439	0.024	0.240	0.599	0.068	0.600	-0.056
Age	0.203	-0.104	0.844	0.022	0.589	0.071	0.014	-0.267
Gestational week	0.753	-0.046	0.627	-0.072	0.058	-0.038	0.167	-0.200
Parity	0.123	-0.201	0.258	-0.150	0.356	0.154	0.817	0.030

BMI, body mass index.

**Table 4:** Yale cohort correlations between galectins and clinical parameters.

	gal-1 (ng/ml)		gal-3 (ng/ml)		gal-9 (ng/ml)	
	*P*	*ρ*	*P*	*ρ*	*P*	*ρ*
Age	0.136	-0.389	0.633	-0.129	0.279	-0.287
Gestational week	0.550	0.161	0.958	-0.015	0.715	-0.099
Parity	0.003	-0.844	0.749	-0.111	0.867	0.060

### 3.5 COVID-19 infection during pregnancy influences the PSG1 levels in maternal circulation

**Figure 3:**
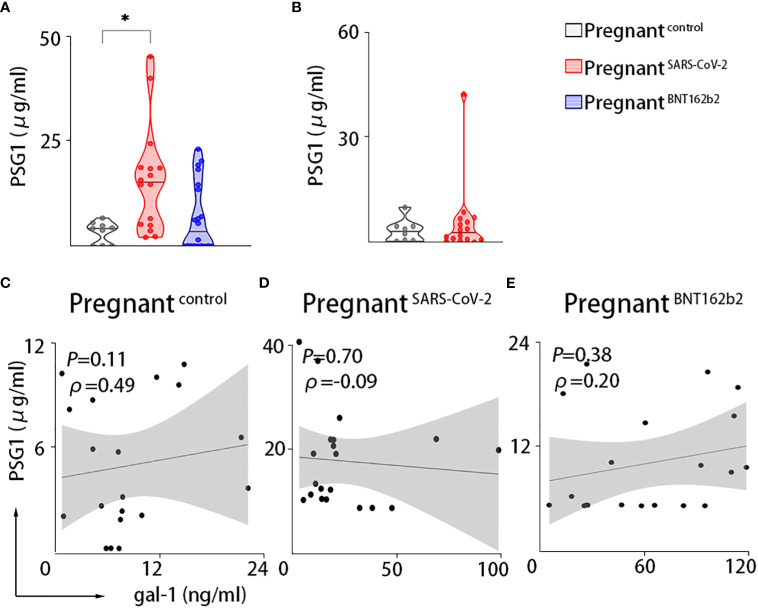
Maternal circulation of PSG1 is only upregulated upon SARS-CoV-2 infection during pregnancy. PSG1. **(A, B)** Maternal circulating levels of PSG1 in the PRINCE cohort **(A)** or the Yale IMPACT cohort **(B)**. **(C–E)** Correlations between serum values of gal-1 and PSG1 in the PRINCE cohort. Pregnant^control^, healthy pregnant women; pregnant^SARS-CoV-2^, pregnant women infected by SARS-CoV-2; pregnant^BNT162b2^, pregnant women vaccinated against SARS-CoV-2. *P < 0.05 as analyzed by Kruskal–Wallis test or the Welch’s t-test.

Because PSG1 is one of the most abundant trophoblastic proteins in maternal serum in the third trimester and binds gal-1 (29), we further examined the PSG1 levels in maternal circulation. Our results in the PRINCE cohort showed that pregnant women infected with SARS-CoV-2 displayed increased levels of PSG1 compared with non-infected pregnant women. A non-significant increase was observed in the vaccinated pregnant women (**Figure 3A**). However, we did not find any correlation between PSG1 and gal-1 (**Figures 3C–E**). The measurement of PSG1 levels in the Yale IMPACT cohort demonstrated similar circulating levels of PSG1 between control pregnant women and pregnant women with SARS-CoV-2 infection (**Figure 3B**).

### 3.6 Transcriptional placental changes of galectins, PSGs, and glycosylation pathways during maternal SARS-CoV-2 infection

**Figure 4:**
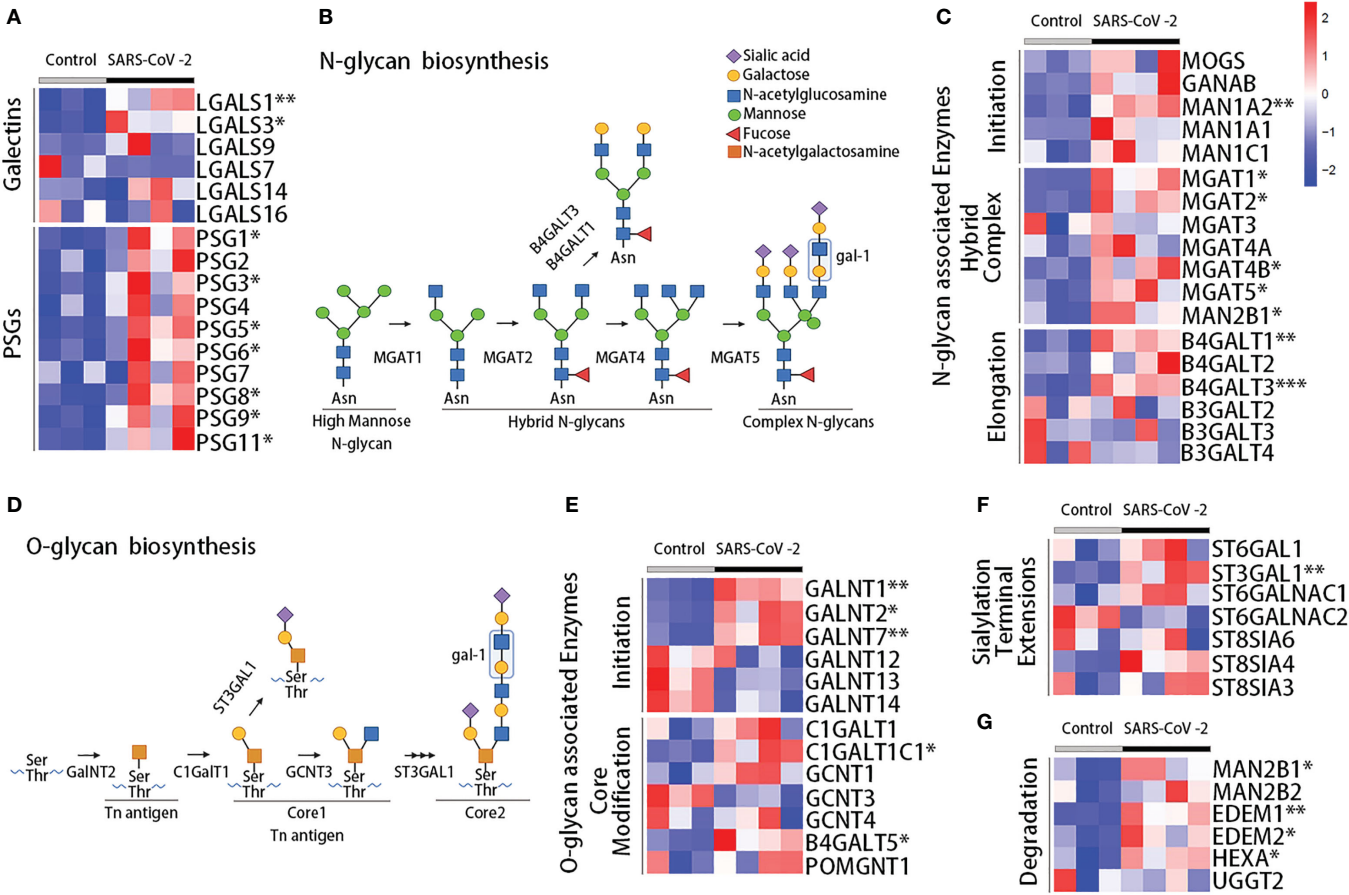
Transcriptome analysis of placenta during acute SARS-CoV-2 infection. **(A)** Heatmap showing the scaled expression of galectins and PSG genes from RNA sequencing data in the placenta of the Yale IMPACT cohort. Enzymes involved in the synthesis of N-glycans **(B)** and O-glycans **(D)**, showing the preferred ligand for gal-1. Heatmaps for the expression of the enzymes involved in the synthesis of N-glycans **(C)** and O-glycans **(E)**, involved in terminal extension **(F)** or glycan degradation **(G)**. Control, healthy pregnant women; SARS-CoV-2, pregnant women infected by SARS-CoV-2. *P < 0.05, **P < 0.01, and ***P < 0.001 as analyzed with the Welch’s t-test.

To further explore the impact of SARS-CoV-2 infection on the placenta compartment, we analyzed RNA sequencing data (GSE171995) of placental villi from the Yale IMPACT cohort. The transcriptomic placental analysis of galectins and PSGs in pregnant women with SARS-CoV-2 infection and matched healthy controls indicated that, consistent with the serum protein measurements, the expression of gal-1 (*LGALS1*, *P* = 0.0077) and gal-3 genes (*LGALS3*, *P* = 0.0152) were increased in the placentas of pregnant women with SARS-CoV-2 infection compared with controls (**Figure 4A**). Moreover, several PSG genes were differentially expressed in the placentas: increased expression of *PSG1* (*P* = 0.0410), *PSG3* (*P* = 0.0456), *PSG5* (*P* = 0.0259), *PSG6* (*P* = 0.0482), *PSG8* (*P* = 0.0355), *PSG9* (*P* = 0.0209), and *PSG11* (*P* = 0.0462) were found in the SARS-CoV-2–infected individuals compared with controls (**Figure 4A**).

Extracellular biological functions of galectins rely on their capacity to bind specific glycans present in proteins on the cell membrane and in the extracellular matrix. Indeed, gal-1 can recognize N- or O-linked glycans containing multiple LacNAc units, which are generated by the enzymes N-acetylglucosaminyltransferases (MGAT genes) or β1,6-N-acetylglucosaminyltransferases (GCNT genes), respectively (**Figures 4B–D**) (6, 49, 50). Poly-LacNAc is also reported as a preferred ligand for gal-3 (51); meanwhile, gal-9 is considered to preferentially bind to internal LacNAc residues of a poly-LacNAc chain (52). We performed differential gene expression analysis to compare the transcriptomic profile of 84 glycoenzymes and generated a hierarchical clustering scheme. A total of 18 glycoenzymes were differentially expressed. Among them, eight are involved in the synthesis and modification of N-linked glycans (**Figure 4C**), five in the synthesis and processing of O-linked glycans (**Figures 4D–E**), and five in terminal extensions of sialylation and its degradation (**Figures 4F, G**). Overall our results showed upregulated expression of glycoenzymes in the placentas of SARS-CoV-2–infected individuals compared with healthy controls, which included the enzymes that take part in the initiation (*MAN1A2*, *P* = 0.0011), branched complex (*MGAT1*, *P* = 0.0131; *MGAT2*, *P* = 0.0142; *MGAT4B*, *P* = 0.0260; *MGAT5*, *P* = 0.0143; *MAN2B1*, *P* = 0.0226), and elongation (*B4GALT1*, *P* = 0.0013; *B4GALT3*, *P* = 0.0004) of N-linked glycans (**Figure 4C**). We also observed an upregulation of the expression of glycoenzymes that take part in the initiation (*GALNT1*, *P* = 0.0015; *GALNT2*, *P* = 0.0172; *GALNT7*, *P* = 0.0023) and core modification (*B4GALT5*, *P* = 0.0302; *C1GALTC1*, *P* = 0.0171) of O-linked glycans (**Figure 4E**) and enzymes linked to terminal extensions of sialylation and degradation including *ST3GAL1* (*P* = 0.0034), *MAN2B1* (*P* = 0.0226), *EDEM1* (*P* = 0.0062), *EDEM2* (*P* = 0.0331), and *HEXA* (*P* = 0.0349) (**Figures 4F, G** **)**. These results suggest an enrichment of N-glycan– and O-glycan–associated enzymes compatible with the gal-1 binding in SARS-CoV-2–infected placentas.

## 4 Discussion

**Figure 5:**
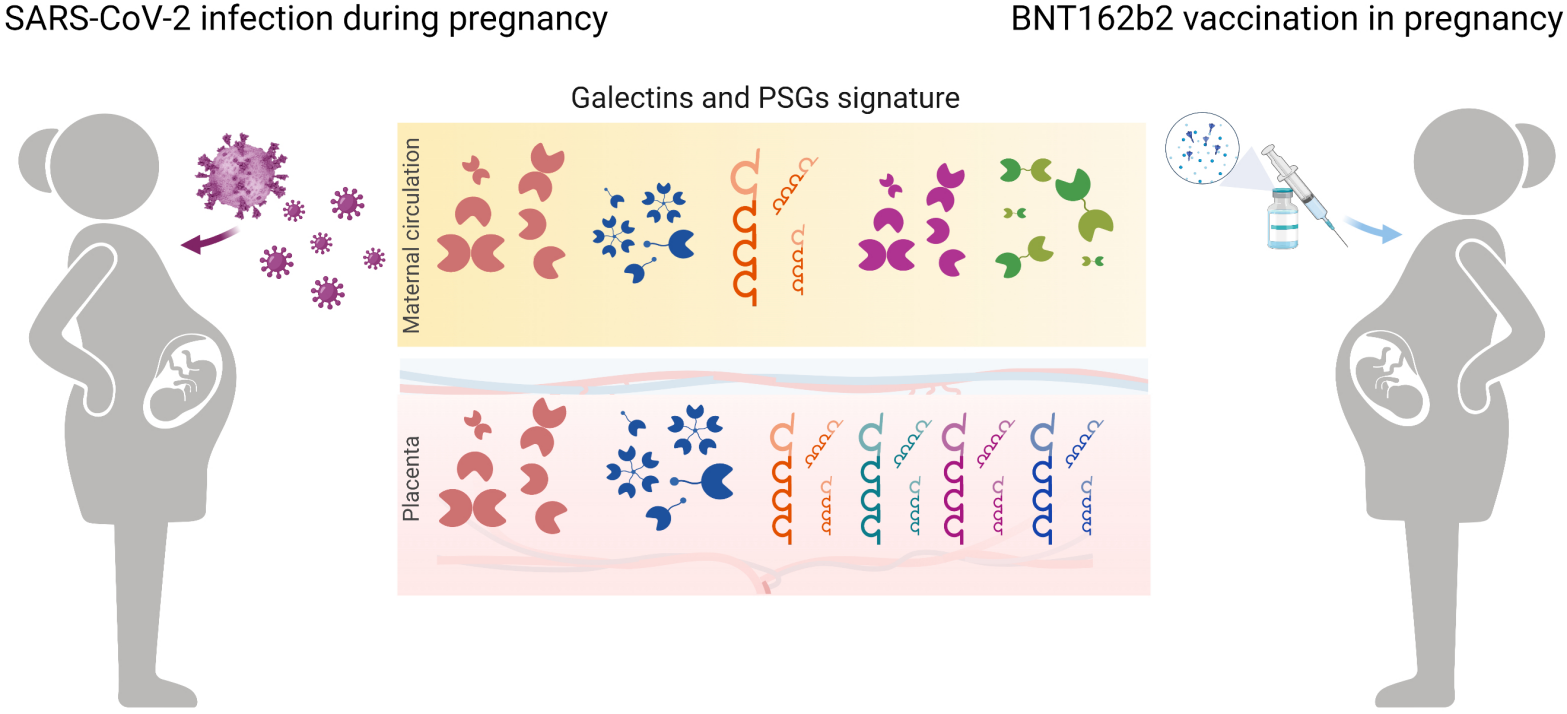
Schematic diagram highlighting galectin and PSGs fingerprints in pregnancy upon SARS-CoV-2 infection and in women subjected to vaccination. In maternal circulation, levels of gal-1 and PSG1 increased during COVID-19 disease in pregnancy. Vaccination with BNT162b2 caused an upregulation of gal-1 circulating levels mimicking the viral infection. During SARS-CoV-2 infection, placental transcription regulation of gal-1 and gal-3 dominates the galectin signature creating a protective microenvironment. In addition, upregulation of PSGs by the trophoblast cells contributes to immune modulation during COVID-19 disease.

SARS-CoV-2 infection leads to a higher risk of severe disease in pregnant than in non-pregnant women. Considering that women of reproductive age make up more than 20% of the global population, studying the effects of SARS-CoV-2 infection and vaccination in this population is of great importance. We measured the concentration of several galectins and PSG1 in the circulation of pregnant women infected with SARS-CoV-2 or vaccinated against SARS-CoV-2 and also explored their placental expression (**Figure 5**). Our results demonstrated a dominant role of gal-1 within the galectin signature in pregnant women upon SARS-CoV-2 infection in both the PRINCE and Yale IMPACT cohorts. Our findings align with previous reports in non-pregnant individuals, indicating that SARS-CoV-2 infection increased gal-1 levels (41, 42). Moreover, one study showed a positive correlation between gal-1; levels of pro-inflammatory cytokines such as Interleukin-1 beta (IL-1β), IL-6, and IL-23; and COVID-19 severity, suggesting that gal-1 acts as an alarmin (42). In non-pregnant patients, gal-3 and gal-9 were found to be increased and correlated with COVID-19 severity (45, 53). However, we did not find increased levels of gal-3 or gal-9 in pregnant women with SARS-CoV-2 infection. This difference in results may be explained, at least, in part, by the fact that most of our cohort patients had mild or non-symptomatic COVID-19 disease. Furthermore, our results showed positive correlations between gal-1 and gal-3 or gal-9 levels only in healthy pregnancies. This positive correlation was expected because successful pregnancy is associated with a rise in maternal circulating levels of these three galectins from the first to the third trimester (28, 54, 55). The observation that only gal-1 was increased as a result of SARS-CoV-2 infection is likely responsible for the observed loss of correlation with the other two galectins in this group of patients.

The immune system plays a key role in mediating a successful pregnancy because a fine balance is necessary to promote maternal tolerance to the allogeneic fetus while protecting both the mother and the fetus from pathogens (56, 57). Several studies demonstrated that the maternal immune response tend to restrain inflammation through regulatory and anti-inflammatory mediators (58, 59), and galectins, especially gal-1, are involved in this process (6). Moreover, gal-1 has been described not only as a damage-associated molecular pattern molecule, amplifying the immune response, but also as a member of a diverse group of mediators collectively referred to as resolution-associated molecular pattern molecules with the capacity to resolve an acute inflammation process by counteracting the synthesis of pro-inflammatory cytokines (60, 61). The cytokine storm in the systemic circulation is responsible for the pathophysiology of SARS-CoV-2 infection and can lead to multi-organ damage (7, 62). Both systemic and inflammatory changes at the maternal–fetal interface were previously described during SARS-CoV-2 infection in pregnancy (48, 63). The analysis of bulk RNA sequencing of placental villi from the Yale IMPACT cohort demonstrated that COVID-19 cases presented increased expression of genes associated with an immune response, indicating a robust response at the maternal–fetal interface upon SARS-CoV-2 infection, even in the absence of localized placental infection (48). Another study evaluated the maternal systemic immune response, finding increased IL-8, IL-10, and IL-15 levels as well as a reduction in T-cell subsets, particularly of T helper cell 1 (Th1) and a subset of CD8^+^ cells characterized by the production of IL-17 (Tc17)-like cells in pregnant women infected with SARS-CoV-2 (63). Gal-1 controls the fate of Th-1 and Th-17 cells through the glycan repertoire expressed by these cells that allow gal-1 binding (64). Therefore, the increase of gal-1 in maternal circulation during COVID-19 could be associated with the observed systemic decrease in Th-1 and Tc17-like cells. Gal-1 also induces apoptosis of activated CD8^+^ T cells (65), which could explain the upregulation of gal-1 in SARS-CoV-2 infection. Thus, the increase of gal-1 observed in pregnant patients with COVID-19 could be related to its anti-inflammatory activity and its potential participation in the control of inflammation and tissue damage resulting from SARS-CoV-2 infection.

In our study, healthy pregnant women exposed to SARS-CoV-2 infection often exhibit simultaneously increased serum concentrations of gal-1 and PSG1. We have previously demonstrated that PSG1 binds to gal-1 and postulated that PSG1 protects gal-1 from inactivation by oxidation in the extracellular environment. Therefore, an increase in both PSG1 and gal-1 may extend the gal-1 activity once it is released from cells into the circulation (29). However, the correlation between these two proteins disappeared in infected pregnant women, likely due to the modest increase of PSG1 relative to gal-1. Unfortunately, we were unable to determine whether the concentration of other PSG family members, which can also interact with gal-1, is also increased in the circulation of SARS-CoV-2–infected pregnant women as suggested by the RNA sequencing data because specific validated ELISAs are only available to measure the concentration of PSG1. Future studies should be performed to determine the mechanism by which syncytiotrophoblast cells, the major cell type that secretes PSG1 into the maternal circulation, regulate PSG1 secretion following maternal SARS-CoV-2 infection. In contrast, PSG1 post-vaccination levels remain unchanged, suggesting that modulation of PSG1 secretion by the syncytiotrophoblast cells requires maternal SARS-CoV-2 infection. In the future, it would be interesting to determine whether an increase in PSG1 concentration is also observed following infection with other viruses that do not infect the PSG1-producing cells and whether this increase in PSG levels may contribute to the establishment of an anti-inflammatory environment.

The extracellular activity of gal-1 relies on its capacity to bind specific glycans. To further investigate the possible role of the gal-1 upregulation during the course of COVID-19, the expression of enzymes involved in relevant N- and O-glycosylation was analyzed in bulk placentas by RNA sequencing. Our results showed that genes involved in the initiation, hybrid complex formation, and elongation of N-glycans and in initiation and core modification of O-glycans were upregulated in patients with COVID-19. Gal-1 and gal-3 can bind β-1,6-GlcNAc–branched N-linked glycans on the cell surface or in secreted glycoproteins, and several enzymes are required for the biosynthesis of these glycotopes (51). Similar results were found for the enzymes involved in O-glycans synthesis, as initiation (GALNT1, GALNT2, and GALNT7) and core modification (C1GALT1C1 and B4GALT5) enzymes were also upregulated during SARS-CoV-2 infection. The addition of α2,6-linked sialic acids to the termini of glycans by ST6GAL1 inhibits the binding of gal-1 (6). However, our results demonstrated similar expression of ST6GAL1 in SARS-CoV-2–infected and control groups. More importantly, we observed an increase of ST3GAL1 expression signatures (responsible for the addition of α2,3-linked sialic acid, which is compatible which gal-1 high-affinity binding) within the placental compartment. These results suggest that both the expression of gal-1 and of the enzymes in the placenta involved in the N- and O-glycan modifications required for gal-1 to exert its function are increased following SARS-CoV-2 infection.

We also explored the galectin fingerprint of healthy pregnant women that were fully vaccinated. Several reports indicated that COVID-19 vaccines are safe for pregnant women and can effectively protect them from getting severe COVID-19 and prevent maternal and fetal mortality (8, 66). Our results provide evidence that the status of the maternal immune system during vaccination modulates the galectin signature and could play a role for protection against COVID-19 disease. For instance, a simultaneous increase of gal-1 and gal-7 accompanied by a decrease of gal-9 in post- vaccination sera derived from pregnant individuals suggests a role of these lectins in the establishment of a protective response against the SARS-CoV-2 virus. Gal-1 is secreted by activated B cells, T cells, and macrophages, and activated lymphocytes secrete gal-7 (67, 68). We hypothesize that the increase of these two galectins in pregnant vaccinated patients could play a role in the effective immune system activation by the COVID-19 vaccine that results in the generation of neutralizing antibodies and virus-specific T-cell responses (69). Elevation of circulating levels of gal-9 in chronic and acute SARS-CoV-2 infection has been reported in non-pregnant individuals, and it was correlated with a severe disease outcome (43). On the basis of our findings, we hypothesized that a lack of increase in gal-9 levels during vaccination likely avoids a pro-inflammatory response during pregnancy vaccination that could result in detrimental effects at the maternal–fetal interface.

In conclusion, our study revealed a clear dominance of gal-1 within the maternal galectin fingerprint during SARS-CoV-2 infection and vaccination in pregnancy. One of the pregnancy-specific gal-1 ligands, PSG1, was also upregulated during the COVID-19 disease, suggesting a potential synergistic immunomodulatory role of these proteins at the maternal–fetal interface. Further work is needed to determine the functional relevance of the galectin signature in maternal circulation during the SARS-CoV-2 infection and vaccination in pregnancy. Specifically, it would be important to understand the molecular mechanism by which gal-1 may control the immune response to a virus during pregnancy.

## Data availability statement

The original contributions presented in the study are included in the article/**Supplementary Material**. Further inquiries can be directed to the corresponding author.

## Ethics statement

The studies involving human participants were reviewed and approved by Hamburg Chamber of Physicians (Ärztekammer Hamburg) approved the study protocol under the registration numbers PV3694 (PRINCE), PV 7312-4710 (PRINCE COVID) and 2021-10647-BO-ff (PRINCE VACCINE). The study was approved by the Yale Institutional Review Board (protocol #2000027690). The patients/participants provided their written informed consent to participate in this study.

## Author contributions

SMB designed the study and secured grant funding. FZ, A-CT, YW, YX, AD, AL-C, PV, EK, CU, JB, PCA, SFF, GSD, and MGG performed experiments and/or analyzed data. The original draft of the manuscript was written by FZ, MGG, and SMB, and further writing, review, and editing were done by all authors. All authors contributed to the article and approved the submitted version.
