# Supplementary material for: A unique maternal and placental galectin signature upon SARS-CoV-2 infection suggests galectin-1 as a key alarmin at the maternal–fetal interface
Source: Front Immunol. 2023 Jul 5;14:1196395. doi: 10.3389/fimmu.2023.1196395 (PMC10354452; doi:10.3389/fimmu.2023.1196395)
Supplement: Supplementary file 1 [file Table_1.docx]

**Supplementary Table 1. Detailed results of transcriptome analysis of placenta during SARS-CoV-2 infection in the Yale IMPACT Cohort as analyzed by Welch’s t test.**

| **Gene symbol** | **Ensemble number** | | **Control** | | **SARS-CoV-2** | | **Log2 Fold change** | **P value** |
| --- | --- | --- | --- | --- | --- | --- | --- | --- |
|  |  |  | **Mean** | **SD** | **Mean** | **SD** |  |  |
| LGALS1 | | ENSG00000100097 | 366.7 | 249.8 | 1816 | 551.2 | 2.3081 | 0.0077 |
| LGALS3 | | ENSG00000131981 | 120.3 | 54 | 612.5 | 212.4 | 2.3481 | 0.0152 |
| LGALS7 | | ENSG00000205076 | 8.59 | 10.4 | 0.125 | 0.25 | -6.1027 | 0.2939 |
| LGALS9 | | ENSG00000168961 | 166.7 | 82.46 | 929.2 | 1103 | 2.4787 | 0.2609 |
| LGALS14 | | ENSG00000006659 | 127.3 | 54.04 | 255.5 | 186.4 | 1.0051 | 0.2685 |
| LGALS16 | | ENSG00000249861 | 10.33 | 7.638 | 6.25 | 9.465 | -0.7249 | 0.5561 |
| PSG1 | | ENSG00000231924 | 647.6 | 602.3 | 6441 | 3447 | 3.3141 | 0.0410 |
| PSG2 | | ENSG00000242221 | 630.5 | 496.1 | 1773 | 757.3 | 1.4916 | 0.0613 |
| PSG3 | | ENSG00000221826 | 673.9 | 442.7 | 3590 | 1834 | 2.4134 | 0.0456 |
| PSG4 | | ENSG00000243137 | 986.7 | 746 | 2690 | 1077 | 1.4469 | 0.0565 |
| PSG5 | | ENSG00000204941 | 393.9 | 148 | 1959 | 789.4 | 2.3142 | 0.0259 |
| PSG6 | | ENSG00000170848 | 336.5 | 128.4 | 993.8 | 426.6 | 1.5623 | 0.0482 |
| PSG7 | | ENSG00000221878 | 73.36 | 41.12 | 117.4 | 75.32 | 0.6784 | 0.3700 |
| PSG8 | | ENSG00000124467 | 112.6 | 41.65 | 394.2 | 162.3 | 1.8077 | 0.0355 |
| PSG9 | | ENSG00000183668 | 561.2 | 241.1 | 2380 | 878.8 | 2.0844 | 0.0209 |
| PSG11 | | ENSG00000243130 | 247.4 | 91.62 | 1185 | 581.8 | 2.2600 | 0.0462 |
| MOGS | | ENSG00000115275 | 22.56 | 15.28 | 112.5 | 87.05 | 2.3181 | 0.1290 |
| GANAB | | ENSG00000089597 | 229.5 | 45.06 | 1466 | 792.5 | 2.6753 | 0.0521 |
| MAN1A2 | | ENSG00000198162 | 386.5 | 138.9 | 1425 | 247.6 | 1.8824 | 0.0011 |
| MAN1A1 | | ENSG00000111885 | 94 | 7.81 | 572.3 | 382.8 | 2.6060 | 0.0877 |
| MAN1C1 | | ENSG00000117643 | 276.4 | 97.63 | 458.7 | 114.8 | 0.7308 | 0.0746 |
| MGAT1 | | ENSG00000131446 | 229.1 | 11.23 | 732.3 | 190.9 | 1.6765 | 0.0131 |
| MGAT2 | | ENSG00000168282 | 58.33 | 13.5 | 260 | 81.74 | 2.1562 | 0.0142 |
| MGAT3 | | ENSG00000128268 | 80.67 | 50.4 | 81.25 | 15.15 | 0.0103 | 0.9861 |
| MGAT4A | | ENSG00000071073 | 127.9 | 111.1 | 348.5 | 229 | 1.4461 | 0.1599 |
| MGAT4B | | ENSG00000161013 | 76 | 21.86 | 241.8 | 85.67 | 1.6697 | 0.0260 |
| MGAT5 | | ENSG00000152127 | 185.7 | 72.15 | 569.8 | 172.1 | 1.6175 | 0.0143 |
| MAN2B1 | | ENSG00000104774 | 150.6 | 43.34 | 271.6 | 54.44 | 0.8508 | 0.0226 |
| B4GALT1 | | ENSG00000086062 | 290 | 170.3 | 1268 | 227.5 | 2.1284 | 0.0013 |
| B4GALT2 | | ENSG00000117411 | 119.7 | 27.23 | 255.8 | 120.3 | 1.0956 | 0.1057 |
| B4GALT3 | | ENSG00000158850 | 71.91 | 15.61 | 234.5 | 30.14 | 1.7053 | 0.0004 |
| B3GALT2 | | ENSG00000162630 | 19 | 16.09 | 16.5 | 16.3 | -0.2035 | 0.8485 |
| B3GALT3 | | ENSG00000225711 | 9.333 | 10.12 | 5 | 9.345 | -0.9004 | 0.5918 |
| B3GALT4 | | ENSG00000235863 | 7.833 | 6.802 | 3.1 | 0.9592 | -1.3373 | 0.3511 |
| GALNT1 | | ENSG00000141429 | 134.3 | 33.17 | 506.8 | 89.09 | 1.9160 | 0.0015 |
| GALNT2 | | ENSG00000143641 | 252.9 | 39.16 | 773.1 | 225.8 | 1.6121 | 0.0172 |
| GALNT7 | | ENSG00000109586 | 71 | 18.73 | 224.5 | 42.47 | 1.6608 | 0.0023 |
| GALNT12 | | ENSG00000119514 | 34.33 | 35.02 | 23.25 | 28.18 | -0.5622 | 0.6773 |
| GALNT13 | | ENSG00000144278 | 86.33 | 86.5 | 28.5 | 20.16 | -1.5989 | 0.3664 |
| GALNT14 | | ENSG00000158089 | 73.33 | 60.71 | 29 | 27.04 | -1.3384 | 0.3346 |
| C1GALT1 | | ENSG00000106392 | 145.5 | 85.89 | 242.9 | 48.52 | 0.7393 | 0.1773 |
| C1GALT1C1 | | ENSG00000171155 | 24.33 | 3.786 | 86.5 | 26.59 | 1.8300 | 0.0171 |
| GCNT1 | | ENSG00000187210 | 69.83 | 32.28 | 114.1 | 10.58 | 0.7084 | 0.1318 |
| GCNT3 | | ENSG00000140297 | 158.8 | 142.5 | 45.57 | 39.51 | -1.8011 | 0.3008 |
| GCNT4 | | ENSG00000176928 | 49.01 | 23.09 | 50.51 | 18.09 | 0.0435 | 0.9307 |
| B4GALT5 | | ENSG00000158470 | 193.7 | 42.92 | 636.7 | 235.7 | 1.7168 | 0.0302 |
| POMGNT1 | | ENSG00000085998 | 169 | 91.01 | 230.6 | 65.94 | 0.4484 | 0.3838 |
| ST6GAL1 | | ENSG00000073849 | 134.4 | 77.55 | 424.8 | 213.2 | 1.6603 | 0.0665 |
| ST3GAL1 | | ENSG00000008513 | 238.7 | 62.88 | 528.9 | 83.17 | 1.9765 | 0.0034 |
| ST6GALNAC1 | | ENSG00000070526 | 48.43 | 44.46 | 13.13 | 9.674 | -1.8830 | 0.3015 |
| ST6GALNAC2 | | ENSG00000070731 | 37.38 | 25.39 | 26.72 | 19.87 | -0.4843 | 0.5817 |
| ST8SIA6 | | ENSG00000148488 | 95.76 | 100.2 | 34.71 | 24.83 | -1.4641 | 0.4027 |
| ST8SIA4 | | ENSG00000113532 | 125.9 | 59.49 | 599.7 | 631.6 | 2.2520 | 0.2306 |
| ST8SIA3 | | ENSG00000177511 | 79.77 | 59.11 | 42.56 | 43.49 | -0.9063 | 0.4157 |
| MAN2B1 | | ENSG00000104774 | 150.6 | 43.34 | 271.6 | 54.44 | 0.8508 | 0.0226 |
| MAN2B2 | | ENSG00000013288 | 243.9 | 110 | 374.4 | 109.2 | 0.6183 | 0.1870 |
| EDEM1 | | ENSG00000134109 | 146.9 | 11.17 | 359.7 | 65.35 | 1.2920 | 0.0062 |
| EDEM2 | | ENSG00000088298 | 49.33 | 18.58 | 113 | 36.51 | 1.1958 | 0.0331 |
| HEXA | | ENSG00000213614 | 260.9 | 114.4 | 529.7 | 68.69 | 1.0217 | 0.0349 |
| UGGT2 | | ENSG00000102595 | 433.6 | 370.3 | 369.6 | 117.9 | -0.2304 | 0.7970 |
